# Baruch Fischhoff: the importance of testing messages

**DOI:** 10.2471/BLT.20.030820

**Published:** 2020-08-01

## Abstract

Why governments need to test their messages on novel coronavirus disease (COVID-19) before disseminating them. Baruch Fischhoff talks to Fiona Fleck.

Q: You started studying risk and human behaviour in the 1970s. What sparked that interest?

A: I majored in mathematics, with a secondary major in psychology and then had the extraordinary good fortune to study and work with pioneers in the field of decision-making, including Daniel Kahneman, Amos Tversky, Paul Slovic, and Sarah Lichtenstein. Eventually that led to the study of the psychology behind judgments and preferences, risk analysis, and risk communication. My professional entry into the field began in the 1970s when my colleagues and I were approached by technologists and engineers trying to understand public resistance to emerging technologies. Initially, they included representatives of companies promoting nuclear power and biotechnologies based on recombinant DNA. They would say, “We have this wonderful technology, but people think it’s too risky because they don’t understand it. You’re psychologists, do something about it.”

Q: What did they want you to do exactly?

A: Help them make the audience understand the great benefits and low risks of their technologies. As they saw it, the public just didn’t get it. It’s a common reaction when people try to communicate and it doesn’t work: they blame their audience. Drawing on psychological theories and methods, we studied how people perceived these new technologies. What we found was that there was very little that the public needed to know that couldn’t be explained in comprehensible terms. However, in order to discover those terms, you needed to listen to the public first and then test your draft messages. There has been a good deal of work on risk communication since then and that work has generated some important insights. For example, the research has found that clearly presented quantitative information can avoid the confusion created by vague quantifiers, such as “likely,” “rare,” and “possible”.

“It’s a common reaction when we try to communicate and it doesn’t work: we blame our audience.”

Q: Can these lessons from the past help us in the response to the COVID-19 pandemic?

A: Absolutely. Just as with the technologists in the 1970s, public health communicators need to know how their audience thinks about new issues, including the COVID-19 pandemic. One of psychology’s strongest results is that we exaggerate how well we understand one another. As a result, we think we know what other people want to hear and we think we are making ourselves abundantly clear. But we’re just guessing, based on our intuitive theories about what other people know and want to know. It’s amazing how few messages are empirically tested, however great the health, economic, and political stakes. People with enormous responsibility just write down something that looks right to them and send it out. Moreover, the most basic message testing costs essentially nothing. All you have to do is to ask a diverse group of people to read a draft message, telling you whatever comes into their minds. There are always surprises. Things that seemed clear to you confuse them. Things that seem clear to them are being interpreted differently than the way you intended. Not testing messages before putting them out is a kind of public health malpractice.

Q: How can the study of human behaviour in relation to risk support health risk communications and messaging regarding preventive measures such as social distancing, mask wearing and handwashing?

A: The discipline of risk communication is straightforward. Gather the evidence regarding the risk, analyse that evidence to determine what people really need to know, draft messages focused on those critical facts, test the draft messages, and repeat as necessary. In the United States of America, we have failed on all three fronts. We have not tested enough people to know the magnitude of the problem – and the risks it represents. We have not synthesized the evidence that people need to be aware of in authoritative, comprehensible form, so people are buffeted by social media rumours and reports of individual studies, sometimes before they have been peer reviewed. Our national academies have a COVID-19 Committee that has done a dozen authoritative and peer-reviewed syntheses of the research on topics, such as the vulnerability of younger people and the effectiveness of handmade fabric face masks. However, the academies cannot track and interpret all of the studies of varying quality that are coming out. So, we don’t get the evidence in the form that we need it. Third, our official communications have often been amateurish.

Q: How do you account for these shortcomings?

A: Basically, we know what to do, but haven’t done it. By ‘we’ I mean our official sources, those responsible for formulating and communicating policies and recommendations that would help us get through crises like the present one. There has been an appreciation of the need to communicate effectively during pandemics for some time and efforts have been made to prepare. For example, in 2016, our national academies held a workshop on capacity building in communication for pandemics. So, we knew what preparations were needed, but were caught unprepared.

Q: What could have been done differently?

A: With a small investment, we could have developed and tested generic messages, ready to adapt to specific situations. For example, we could have been ready to explain the epidemiological models that are so essential for understanding what’s happening. We could have been ready to explain how diseases spread, and the risk of transmission, including to people we may never even see. We could have reduced the confusion over mask recommendations meant to protect the people who wear them and the people who are near them. Instead, we have improvised, serving the public poorly and losing its trust. Science and health reporters in the responsible news media have done important work making sense of the science and the controversies. However, the media work in a piecemeal way. They are not a substitute for succinct credible official guidance, reflecting policies sensitive to the needs of our diverse populations.

Q: Can you give examples of risk assessment work related to health that you have done with policy-makers?

A: Between 2007 and 2011, I worked with the Food and Drug Administration (FDA) as chair of its statutory Risk Communication Advisory Committee. We helped the FDA implement its strategic plan for risk communication, proposed fast, efficient messages testing procedures, and recommended policies for “emerging events.” I also helped the FDA to rework how it reports approval decisions for pharmaceuticals and biologics. The FDA subsequently created a Benefit-Risk Framework, which is an excellent example of how to communicate complicated decisions. The framework uses a standard format, structured around key issues. It reports evidence *and* uncertainties, and sets out the rationale for its regulatory decisions. It also identifies ways to make approved products even better. There is no reason why we could not use that kind of framework to organize and communicate the evidence regarding other health issues. It’s not expensive, it uses staff time efficiently, and it reduces the health, economic, and political risks of failing to address the public’s concerns.

“Not testing messages before putting them out is a kind of public health malpractice.”

Q: What is your advice to governments when preparing and communicating their guidance?

A: Avoid long to-do lists with no organizing rationale. Also, focus on the decisions that people face in their daily lives, so that the information communicated is relevant. For example, when I am making a decision how to respond to the risks posed by COVID-19, I ask myself, “How much disease is out there?”, “How close am I going to get to it?”, “What happens if I am exposed?”, and “How can I reduce the risks, at reasonable cost?” Fortunately, our county health department provides good information on disease patterns in our area. I am also fortunate to have enough control over my life that I can limit my exposures. I have access to good health care. I don’t have relatives in care institutions or in prison, whom I need to worry about. So, I just need to worry about the risk to myself and my wife. With respect to, “How can I control things?”, my personal risk analysis is fairly simple: what can I most easily do to put a barrier between myself and other people in case they are shedding virus or I am without knowing it? People will reach different conclusions, depending on their circumstances. However, they can all use communications that present the basic facts, and uncertainties, in a simple, usable, respectful way. Also, I would advise governments to communicate in a way that takes accounts of people’s beliefs. People, including experts, process new information in terms of their mental model, which is comprised of their assumptions, beliefs, experiences, and biases about the world. Communicators need to be aware of these models in crafting their messages.

Q: How well has the World Health Organization (WHO) communicated the risks relating to COVID-19?

A: WHO, as I see it, treats its audience as responsible individuals capable of understanding things and making responsible choices. When I go to the WHO website – and I also send people there – I see pictures of diverse people in settings that would be familiar to many. My sense is that someone who goes there would say, “The people who put this together care about people like me. They know how we live. They are trying to help us solve our problems.” WHO has an advantage in having a very diverse workforce, and you can tell that people from different backgrounds have a hand in the crafting messages.

Q: How has politics informed messaging around COVID-19?

A: As you might expect, it has introduced bias and spin. Wise politicians realise the limits to their knowledge and their ability to spin things in the real world. This is a disease, it doesn’t care what we think and say, it only cares about what we do. If politicians have a short-term agenda and cherry pick the data, or find a scientist who happens to agree with them, they might win in the short run, but they leave themselves vulnerable in the long run. Wise policy advisers encourage policy-makers to respect the science, and, of course to communicate evidence-based messages as effectively as possible.

